# Prognostic impact of lymphadenectomy in clinically early stage malignant germ cell tumour of the ovary

**DOI:** 10.1038/bjc.2011.267

**Published:** 2011-07-19

**Authors:** H Mahdi, R E Swensen, R Hanna, S Kumar, R Ali-Fehmi, A Semaan, H Tamimi, R T Morris, A R Munkarah

**Affiliations:** 1 Department of Obstetrics and Gynecology, University of Washington School of Medicine, Seattle, WA 98195, USA; 2 Division of Gynecologic Oncology, University of North Carolina, Chapel Hill, NC 27599, USA; 3 Division of Gynecologic Oncology, Mayo Clinic, Rochester, MN 55905, USA; 4 Department of Pathology, Wayne State University School of Medicine, Detroit, MI 48201, USA; 5 Division of Gynecologic Oncology, Barbara Ann Karmanos Cancer Institute, Wayne State University School of Medicine, Detroit, MI 48201, USA; 6 Department of Women's Health Services, Henry Ford Health System, 2799 West Grand Boulevard, Detroit, MI 48201, USA

**Keywords:** lymphadenectomy, survival, clinical stage I, malignant germ cell tumour, ovary

## Abstract

**Background:**

The aim of this study was to determine the impact of lymphadenectomy and nodal metastasis on survival in clinical stage I malignant ovarian germ cell tumour (OGCT).

**Methods:**

Data were obtained from the National Cancer Institute registry from 1988 to 2006. Analyses were performed using Student's *t*-test, Kaplan–Meier and Cox proportional hazard methods.

**Results:**

In all, 1083 patients with OGCT who have undergone surgical treatment and deemed at time of the surgery to have disease clinically confined to the ovary were included 590 (54.48%) had no lymphadenectomy (LND−1) and 493 (45.52%) had lymphadenectomy. Of the 493 patients who had lymphadenectomy, 441 (89.5%) were FIGO surgical stage I (LND+1) and 52 (10.5%) were upstaged to FIGO stage IIIC due to nodal metastasis (LND+3C). The 5-year survival was 96.9% for LND−1, 97.7% for LND+1 and 93.4% for LND+3C (*P*=0.5). On multivariate analysis, lymphadenectomy was not an independent predictor of survival when controlling for age, histology and race (HR: 1.26, 95% CI: 0.62–2.58, *P*=0.5). Moreover, the presence of lymph node metastasis had no significant effect on survival (HR: 2.7, 95% CI: 0.67–10.96, *P*=0.16).

**Conclusion:**

Neither lymphadenectomy nor lymph node metastasis was an independent predictor of survival in patients with OGCT confined to the ovary. This probably reflects the highly chemosensitive nature of these tumours.

Malignant germ cell tumours of the ovary (OGCT) are rare, accounting for only 7% of ovarian cancers (Koonings *et al*, 1989). Ovarian germ cell tumour encompass tumours with multiple histologic patterns, and variable biologic behaviours and are highly chemosenstive (Serov *et al*, 1973; Scully and Sobin, 1999). These tumours are rapidly growing, predominately unilateral and confined to one ovary in two thirds of cases (Gershenson, 2007). They principally affect girls and young women and cure rates are relatively high; therefore, fertility preservation is an important factor to consider in any treatment approach. The standard surgical approach for patients with clinical stage I OGCT includes unilateral salpingo-oophorectomy with surgical staging (Kurman and Norris, 1977; Peccatori *et al*, 1995; Gershenson, 2007).

Comprehensive surgical staging in ovarian cancer has traditionally included peritoneal cytology, inspection and palpation of abdominopelvic contents, pelvic and para-aortic lymph node dissection, omentectomy, and peritoneal biopsies or removal of any suspicious lesion (ACOG, 2002). Some studies have shown that performing a lymphadenectomy has a significant impact on survival in early stage epithelial ovarian cancer (Petru *et al*, 1994; Cass *et al*, 2001; Chan *et al*, 2007; Rouzier *et al*, 2010). However, these conclusions have not been consistent for all histologic types. Schmeler *et al* (2010) reported no cases of nodal metastasis in patients with clinical stage I mucinous tumour of the ovary and found no significant difference in progression-free survival and overall survival between patients who underwent lymphadenectomy and those who did not. Brown *et al* (2009) suggested that lymphadenectomy might be omitted when staging patients with sex cord-stromal tumours of the ovary.

Many young patients with OGCT present with acute abdominal pain and are suspected to have an acute but nonmalignant diagnosis (bleeding haemorrhagic cyst, ectopic pregnancy, ruptured appendix) that necessitates surgical intervention. As a result, many are incompletely staged at the time of the primary surgery due to either the absence of intraoperative pathologic diagnosis or performance of surgery by a surgeon who lacks the expertise to complete a surgical staging (Gershenson, 2007). This represents a challenging management decision after the final diagnosis is confirmed as to whether or not a second operative procedure for more comprehensive surgical staging should be performed. A recent Children Intergroup study has raised a question regarding the extent of surgical staging needed in children with OGCT. The authors suggested that a more conservative surgical staging approach that includes removing the affected ovary, palpating the retroperitoneal lymph nodes and only excising firm or enlarged nodes and any suspicious lesions in the abdomen and pelvis may be sufficient and an adequate substitute to a more comprehensive lymphadenectomy in their patient population (Billmire *et al*, 2004).

Using the large population-based database maintained by the National Cancer Institute, the objective of this retrospective study was to evaluate the survival impact of lymphadenectomy and nodal metastasis in women diagnosed with clinically apparent early stage OGCT.

## Materials and methods

Subjects with a diagnosis of OGCT grossly confined to the ovary during the period from 1 January 1988 to 31 December 2006 were identified using the Surveillance, Epidemiology and End Results (SEER) program of the United States National Cancer Institute. The SEER database provides information on the disease stage based on clinical, intraoperative and pathological findings sufficient to give a fair estimation of the disease extent. In this study, we labelled patients as having ‘clinical stage I’ disease if they have undergone surgical treatment with intraoperative findings, suggesting that the disease is clinically confined to the ovary (Morice *et al*, 2003; Kumar *et al*, 2008; Schmeler *et al*, 2010). Patients were divided into three cohorts: clinical stage I (patients with disease grossly confined to the ovary and no lymphadenectomy) (LND−1), FIGO stage I (patients with disease grossly confined to the ovary who underwent lymphadenectomy with histologically negative nodes) (LND+1) and FIGO stage IIIC (patients with disease grossly confined to the ovary who underwent lymphadenectomy with histologically positive nodes) (LND+3C). Histology codes (ICD-O3) were used to identify various types of OGCT and divided into three categories: dysgerminoma (D), malignant teratoma (MT) and mixed germ cell tumour with pure nondysgerminoma cell tumour (MGCT/PNCT) as previously published (Kumar *et al*, 2008). The inclusion criteria were clinical stage I, surgical treatment, known age, known histology type and active follow-up. Patients with clinical stage other than stage I were excluded. Other exclusion criteria were patients with unknown age, unknown status of lymph node dissection, absence of surgical resection of the tumour and a diagnosis by autopsy or death certificate. Both the FIGO stage and clinically apparent stage were determined according to the SEER guidelines. Patients were categorised in the lymphadenectomy group if any lymph nodes were recovered. Demographic, clinico-pathologic, treatment and survival information were extracted using the ‘Case Listing’ option of the SEER Stat 6.4 software. The SEER database does not include any information regarding chemotherapy. Postoperative surveillance refers to postoperative observation only with no chemotherapy treatment after surgery.

Associations between categorical covariates were assessed using *χ*^2^-tests. Group differences were assessed using Student's *t*-test. Survival curves were estimated using the Kaplan–Meier method. Comparisons were made using log-rank statistics. Cox proportional hazard (PH) regression was used to adjust for age, race, histology and lymph node metastasis. All *P*-values reported are raw values for single comparisons and a *P*-value of <0.05 was considered statistically significant. STATA 10.0 program (College Station, TX, USA) was used for the analysis of the data.

## Results

Out of the 1083 patients who met the inclusion criteria, lymphadenectomy was performed in 493 patients (45.52%). In the lymphadenectomy group, 441 (89.5%) were node negative (FIGO stage I) and 52 (10.5%) were node positive and were, therefore, upstaged to FIGO stage IIIC. The mean age was similar in all groups (Table 1). The median and mean number of nodes recovered in those who had lymphadenectomy was 8 and 11, respectively (range 1–47). Among patients who had nodal metastasis, the median and mean number of positive nodes was 1 and 2, respectively (range 1–15). The median number of nodes recovered in patients who had negative nodes was 8, compared with 7 in those who had positive nodes (*P*=0.27).

White patients were more likely to undergo lymphadenectomy compared with African-American patients (47% *vs* 35.1%, *P*=0.02). The frequency of lymphadenectomy also varied by histologic subtypes with the highest frequency seen in patients with dysgerminoma (62.4%), followed by MGCT/PNDCT (44.1%) and MT (34.5%, *P*<0.001). The rate of lymph node metastasis was the highest in dysgerminoma (Table 1). Women with bilateral tumour (stage IB) were 1.4 times more likely to have lymphadenectomy compared to those with unilateral tumours (stage IA) (63.4 *vs* 44.3, *P*=0.04).

**Figure 1:**
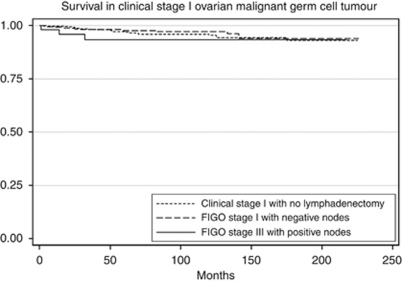
Survival comparison among clinical stage I OGCT (LND−1), histologically node negative stage I OGCT (LND+1) and histologically node positive stage IIIC OGCT (LND+3C). Kaplan–Meier curves for the difference in overall survival between LND−1, LND+1 and LND+3C. Overall 5-year survival was 96.9% for LND−1, 97.7% for LND+1 and 93.4% for LND+3C (*P*=0.5).

**Figure 2:**
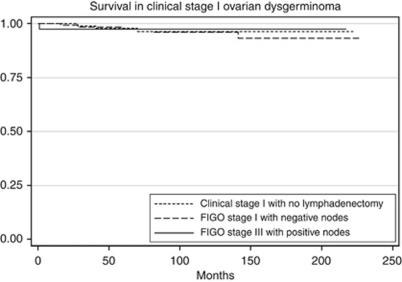
Survival comparison among clinical stage I dysgerminoma (LND−1), histologically node negative stage I dysgerminoma (LND+1) and histologically node positive stage IIIC dysgerminoma (LND+3C). Kaplan–Meier curves for the difference in overall survival between LND−1, LND+1 and LND+3C. Overall 5-year survival was 97.9% for LND−1, 97.5% for LND+1 and 97.5% for LND+3C (*P*=0.92).

**Figure 3:**
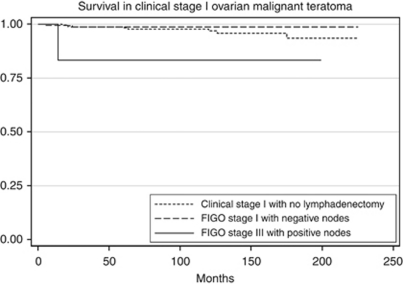
Survival comparison among clinical stage I MT (LND−1), histologically node negative stage I MT (LND+1) and histologically node positive stage IIIC MT (LND+3C). Kaplan–Meier curves for the difference in overall survival between LND−1, LND+1 and LND+3C. Overall 5-year survival was 96.8% for LND−1, 98.6% for LND+1 and 83.3% for LND+3C (*P*=0.38).

**Figure 4:**
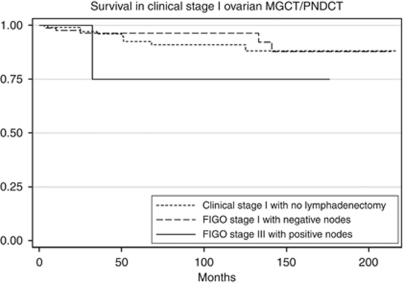
Survival comparison among clinical stage I MGCT/PNDCT (LND−1), histologically node negative stage I MGCT/PNDCT (LND+1) and histologically node positive stage IIIC MGCT/PNDCT (LND+3C). Kaplan–Meier curves for the difference in overall survival between LND−1, LND+1 and LND+3C. Overall 5-year survival was 92.4% for LND−1, 96.3% for LND+1 and 75% for LND+3C (*P*=0.1).

The 5-year survival was 96.9% for LND−1, 97.7% for LND+1 and 93.4% for LND+3C (*P*=0.5; Figure 1). Subgroup analysis was performed to delineate the impact of lymph node involvement on survival across all the histology types. There was no difference in the survival of patients with dysgerminoma when stratified by lymphadenectomy and lymph node status (Table 2; Figure 2). For patients with MT or MGCT/PNDCT, the survival was lower for patients with FIGO stage IIIC disease, but the difference was not statistically significant (Table 2; Figures 3 and 4). When stratified based on the extent of lymphadenectomy, the 5-year survival for those who had <10 *vs* ⩾10 nodes removed was 96% and 98%, respectively (*P*=0.38) compared with 97% for those who did not have lymphadenectomy (*P*=0.64).

In multivariate analysis, lymphadenectomy was not an independent predictor of survival (HR: 1.26, 95% CI: 0.62–2.58, *P*=0.5). This finding was not altered when only patients with lymphadenectomy were included and the analysis performed based on extent of lymph node dissection (HR: 0.8, 95% CI: 0.3–2.4, *P*=0.69). The presence of lymph node metastasis in the lymphadenectomy group had no statistically significant effect on survival after controlling for age, race and histology (HR: 2.7, 95% CI: 0.67–10.96, *P*=0.16); however, the significance of this finding should be interpreted with caution in view of the small number of lymph node positive patients especially in the MT and MGCT/PNDCT subgroups.

## Discussion

The standard therapy for stage I OGCT includes fertility sparing surgery with unilateral salpingo-oophorectomy, and surgical staging followed by postoperative chemotherapy. Before the introduction of combination chemotherapy, the survival of patients with early stage OGCT was dismal with only 5–20% of patients survived after surgery alone, or with postoperative radiation or single-alkylating agent chemotherapy (Gershenson, 1994; Pectasides *et al*, 2008). By the 1970s, the evolution of cisplatin-based chemotherapy led drastic improvement in survival of those patients. The rate of sustained remission after three cycles of chemotherapy with bleomycin, etoposide and cisplatin (BEP) has been found to exceed 95% in multiple reports (Gershenson *et al*, 1990; Williams *et al*, 1994; Dimopoulos *et al*, 2004).

Knowledge of accurate disease stage and thus performing a lymphadenectomy may be of value *in situations* where chemotherapy can be omitted such as stage IA dysgerminoma and stage IA grade I MT. On the other hand, our data do not support the hypothesis that lymphadenectomy has a therapeutic benefit by itself since neither performing a lymph node dissection nor the number of lymph nodes removed had an impact on survival in the study population. These findings suggest that lymph node dissection may not add value in patients who will need adjuvant chemotherapy treatment based on tumour histology or stage. In a Gynecologic Oncology Group trial, 93 patients with OGCT (60 stage I, 10 stage II, 23 stage III) received three cycles of BEP postoperatively. Of them, 96% (91 out of 93) remained free of disease with median follow-up of 38.6 months (4–90.3 months) (Williams *et al*, 1994). All patients underwent surgical resection of the tumour and had comprehensive surgical staging, including biopsy of abnormally palpable nodes; however, routine pelvic and para-aortic nodes sampling was not mandated. In a prospective study by Dimopoulos *et al* (2004), 48 patients (31 stage I, 3 stage II, 12 stage III and 2 stage IV) were treated with either three cycles or four cycles of BEP depending on stage and completeness of surgical resection. With median follow-up of 5 years, 96% of patients were disease free and 100% patients with stage I/II disease or dysgerminoma remained disease free. Some patients in that study underwent biopsy of suspicious pelvic or para-aortic nodes; however, routine lymphadenectomy was not part of the initial staging procedure.

Pelvic and para-aortic lymphadenectomy is a procedure with relatively low risks especially if performed by an adequately trained surgeon; however, it is not completely void of complications. In a randomised trial by Panici *et al* (2005) in patients with epithelial ovarian cancer, higher incidence of perioperative and late complications were noted in patients who received systemic lymphadenectomy compared with the control arm (28% *vs* 18%, respectively, *P*=0.014). Additionally, the author reported a 90-min longer median operating time (*P*<0.001), 350 ml higher blood loss (*P*<0.001) and 12% increase in blood transfusion (*P*=0.006) in the lymphadenectomy group.

The poor sensitivity of intraoperative assessment of the retroperitoneal lymph nodes by inspection and palpation specifically in patients with epithelial ovarian and endometrial cancers is one of the reasons that retroperitoneal lymph node dissection is used for disease staging. The data related to clinical assessment of lymph nodes in germ cell tumours is limited, and come mainly from the paediatric literature. In a study of the Pediatric Oncology Groups/Children's Cancer Group (POG/CCG) on children with OGCT, Billmire *et al* (2004) suggested that an intraoperative clinical assertion of grossly normal lymph nodes is accurate in ruling out metastasis. On the other hand, a clinical suspicion of lymph node metastasis needs histologic confirmation. In fact, all lymph node specimens resected from patients with clinically normal lymph nodes were found free of disease; whereas only 19 of 49 (41%) patients with clinically suspicious nodes had evidence of lymphatic metastasis on histologic evaluation. In another POG/CCG intergroup study that included 57 patients with OGCT, standard staging lymphadenectomy was only performed in three patients (2%), and biopsy of enlarged nodes was performed in another 18 patients (32%). The authors found no histologic evidence of lymphatic metastasis in any of the 23 lymph node samples resected, including 10 macroscopically suspicious nodes (Rogers *et al*, 2004). In a separate study of testicular germ cell tumours by POG/CCG, 63 patients with stage I testicular germ cell tumour were treated with surgery alone followed by observation. Six patients, who had regional recurrences, had not undergone standard retroperitoneal lymph node sampling or dissection at primary surgery. All six were successfully salvaged with chemotherapy (Schlatter *et al*, 2003). These findings from POC/CCG intergroup raise the question of whether sampling only suspicious lymph nodes has the same predictive value and can substitute lymphadenectomy in OGCT patients where postoperative surveillance without adjuvant chemotherapy is planned (patients with stage IA dysgerminoma and stage IA grade I MT). Further clinical studies and trials, especially in adults, are needed to validate the safety and efficacy.

Controversies still exist regarding the management of young patients incidentally diagnosed with OGCT who were incompletely staged at the time of primary surgery. The data are limited regarding the impact of surgical restaging on prognosis or subsequent management. The POC/CCG literature suggests that there is no need for surgical restaging. An alternative approach proposed by Gershenson (2007) is to use CT imaging and tumour markers to guide the decision whether to perform surgical restaging or not.

In this study, race impacted the chance of having lymphadenectomy. The proportion of white patients who underwent lymphadenectomy was significantly higher than that of African-American patients. Prior reports comparing the treatment difference between white and African-American patients with ovarian cancer suggest that African Americans were less likely to receive the standard treatment. Parham *et al* (1997) reported that African Americans with advanced ovarian cancer were less likely to receive primary surgery or combined treatment and twice as likely as whites not to receive the standard therapy. Furthermore, Chan *et al* (2008) reported that a significantly lower proportion of African-American women with early stage epithelial ovarian cancer underwent lymphadenectomy compared with whites. Further studies are warranted to explore the factors behind the racial discrepancy in the extent of medical care.

A major limitation of this study is the lack of information on the adjuvant chemotherapy after initial surgery. Other limitations include lack of central pathology review, information about recurrence and subsequent therapies. The strengths of this study include the fact that this is one of the larger studies on early stage OGCT evaluating the impact of lymphadenectomy on survival. Because the SEER cancer registries are consistent in representative regions throughout the country, the results from this population-based study can be generalised to the entire US population with less potential selection and surveillance biases associated with single institution studies (Hankey *et al*, 1999; Chan *et al*, 2008). SEER data have been shown to be reliable in reporting surgical procedures and adjuvant therapy (Cooper *et al*, 2002; Virnig *et al*, 2002).

In summary, this study found that the addition of lymphadenectomy did not provide survival benefit in patients whose disease was clinically confined to the ovary. Our data were extracted from 1988 where the specific cisplatin-based chemotherapy was fairly the standard regimen. Some data from the paediatric literature suggest that systematic lymphadenectomy for staging purpose can be replaced by biopsying suspicious lymph nodes in the setting of OGCT. While our study is limited by its retrospective nature and lack of information on chemotherapy, one can easily conclude that lymphadenectomy does not add a survival benefit in patients who need postoperative chemotherapy based on histologic type or tumour extension outside the capsule of the ovary. On the other hand, lymphadenectomy may be considered in patients with stage IA dysgerminoma or stage IA grade I MT where postoperative chemotherapy can be omitted. The rarity of this disease makes randomised trials to answer this question very hard if not impossible. An alternative would be to complete multi-institutional and cooperative prospective data collection that can address many of the limitations mentioned above and inherent to retrospective studies.

## Figures and Tables

**Table 1:** Key variables in clinical stage I OGCT patients

**Variable**	**LND-1^a^ (%)**	**LND+1^b^ (%)**	**LND+3C^c^ (%)**	***P***
*Age*				
Mean	24.2	25.2	22.7	NS
Median	22	23	21.5	
				
*Race*				
W	437 (52.97)	343 (41.58)	45 (5.45)	0.07
AA	72 (64.9)	35 (31.5)	4 (3.6)	
O	81 (55.1)	63 (42.9)	3 (2.0)	
				
*Clinical stage*				
Stage IA	434 (55.7)	316 (40.6)	29 (3.7)	0.009
Stage IB	11 (36.6)	17 (56.7)	2 (6.7)	
Stage IC	105 (49.1)	91 (42.5)	18 (8.4)	
Stage I NOS	40 (66.7)	17 (28.3)	3 (5.0)	
				
*Grade MT only*				
Grade I	92 (72.4)	33 (26.0)	2 (1.6)	0.052
Grades II–IV	168 (60.2)	107 (38.4)	4 (1.4)	
				
*Histology*				
Dysgerminoma	133 (37.6)	181 (51.1)	40 (11.3)	<0.001
MT	338 (65.5)	171 (33.1)	7 (1.4)	
MGCT/PNDCT	119 (55.8)	89 (41.8)	5 (2.4)	
				
*Status*				
Alive	569 (96.4)	429 (97.3)	49 (94.2)	0.45
Dead	21 (3.6)	12 (2.7)	3 (5.8)	

Abbreviations: MGCT/PNCT=mixed germ cell tumour with pure nondysgerminoma cell tumour; MT=malignant teratoma; OGCT=ovarian germ cell tumour.

a LND−1=patients with clinical stage I and no lymphadenectomy.

b LND+1=patients with lymphadenectomy and histologically negative nodes.

c LND+3=patients with lymphadenectomy and histologically positive nodes.

**Table 2:** 5-Year survivals in clinical stage I OGCT patients by stage and histology

**Variable**	***N***	**LND-1 (%)**	**LND+1 (%)**	**LND+3C (%)**	***P*-value**
Overall	1083	96.9	97.7	93.4	0.5
					
*Clinical stage*					
Stage IA	779	97.3	98.9	91.7	0.18
Stage IB	30	90	100	100	0.50
Stage IC	214	97.4	94	94.4	0.86
Stage I NOS	60	94.2	94.1	100	0.59
					
*Histology*					
Dysgerminoma	354	97.9	97.5	97.5	0.99
MT	516	98.3	98.6	83.3	0.10
MGCT/PNDCT	213	92.4	96.3	75	0.38
MT+MGCT/PNDCT	729	96.6	97.8	79.5	0.053

Abbreviations: MGCT/PNDCT=mixed germ cell tumour with pure nondysgerminoma cell tumour; MT=malignant teratoma; OGCT=ovarian germ cell tumour.
